# Supplementary material for: Sex- and species-associated differences in complement-mediated immunity in humans and rhesus macaques
Source: mBio. 2024 Feb 22;15(3):e00282-24. doi: 10.1128/mbio.00282-24 (PMC10936177; doi:10.1128/mbio.00282-24)
Supplement: Supplemental Material — Supplemental figures, tables, and legends. [file mbio.00282-24-s0004.pdf]

Supplemental Figures and Legends	
Supplemental Figure 1	Conserved IgG binding sites in human and rhesus C1q- subunit B
Supplemental Figure 2	Comparison of levels of complement proteins in rhesus sera by sex
Supplemental Figure 3	Binding of anti-HIV-1 antibodies to HIV-1 envelope glycoproteins
Supplemental Figure 4	Gating strategy for lytic activity of Fc-engineered antibodies
Supplemental Figure 5	Fold change of lytic activity of Fc-engineered antibodies
Supplemental Figure 6	Gating strategy for Complement-aided Antibody-dependent Phagocytosis (C'ADCP)
**Supplemental Tables and Legends**	
Supplemental Table 1	Human and Rhesus Serum sample cohort
Supplemental Table 2	Antigens used in multiplex assay
**Supplemental Data Files**	
Data File 1	Level of Complement proteins in human and rhesus sera
Data File 2	Antibody-dependent deposition of complement protein C1q by Human and Rhesus serum
Data File 3	Antibody-dependent deposition of complement protein C3b by Human and Rhesus serum

```
P02746          MMMKIPWGSIPVLMLLLLLGLIDISQAQLSCTGPPAIPGIPGIPGTPGPDGQPGTPGIKG
G7MHG1          MMMKILWGSIPVLMLLLLLGLLDVSWAQGSCTGPPAIPGTPGIPGTPGSDGQPGTPGIKG
                ***** ***************:*:* ** ********** ********.***********

P02746          EKGLPGLAGDHGEFGEKGDPGIPGNPGKVGPKGPMGPKGGPGAPGAPGPKGESGDYKATQ
G7MHG1          EKGLPGLAGDHGEFGEKGDPGIPGNPGKVGPKGPMGPKGGPGAPGAPGPKGESGDYKATQ
                ************************************************************

P02746          KIAFSATRTINVPLRRDQTIRFDHVITNMNNNYEPRSGKFTCKVPGLYYFTYHASSRGNL
G7MHG1          KIAFSATRTINTPLRRDQTIRFDHVITNMNNNYEPRSGKFTCRVPGLYYFTYHASSRGNL
                ***********.******************************:*****************

P02746          CVNLMRGRERAQKVVTFCDYAYNTFQVTTGGMVLKLEQGENVFLQATDKNSLLGMEGANS
G7MHG1          CVKLMRGRERPQKVVTFCDYAYNTFQVTTGGMVLKLEQGENVFLQATDKNSLLGMEGANS
                **:*******.*************************************************

P02746          IFSGFLLFPDMEA
G7MHG1          IFSGFLLFPDVEA
                **********:**
```

P02746= Human C1qB,
G7MHG1= Rhesus C1qB

Supplemental Figure 1: Conserved IgG binding sites in human and rhesus C1q- subunit B. Sequence alignment between human and rhesus C1q- subunit B using the software ClustalW2.1. The residues in the globular head (cantaloupe) of human C1q- subunit B expected to bind human IgG are marked in red.

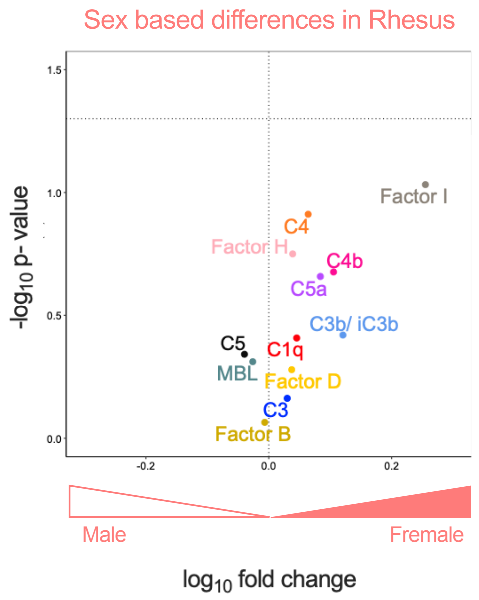

Supplemental Figure 2: Comparison of levels of complement proteins in rhesus sera by sex. Volcano plots of significance (Welch's t-test) and mean fold change of complement protein levels in rhesus females and males. Dotted horizontal line indicates unadjusted p=0.05.

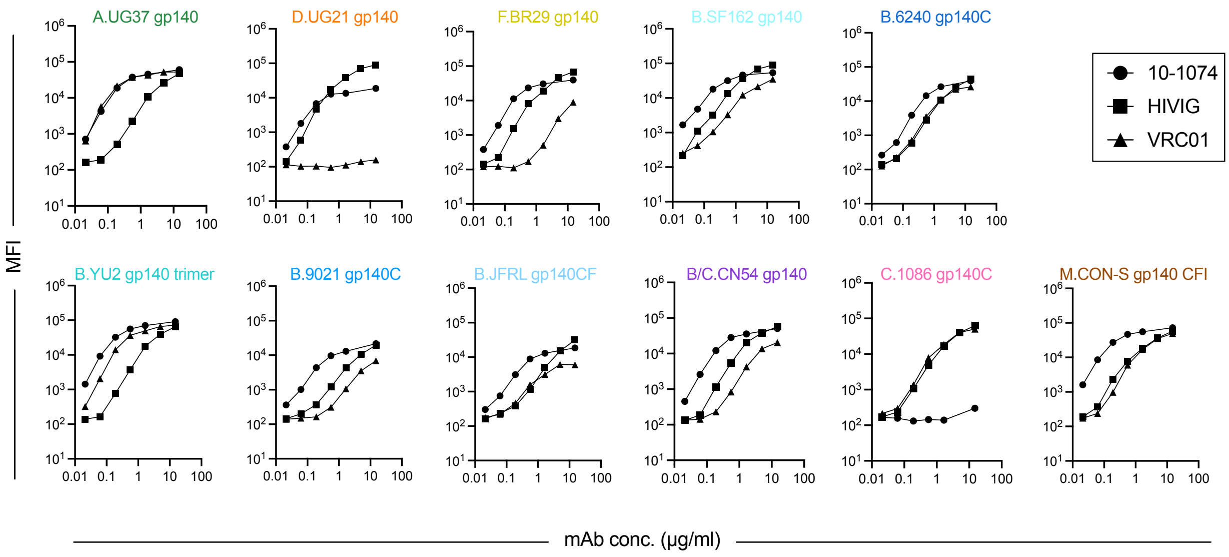

Supplemental Figure 3: Binding of anti-HIV-1 antibodies to HIV-1 envelope glycoproteins. The binding curves for antibodies 10-1074 (circle), HIVIG (square) and VRC01 (triangle) to bind to HIV-1 envelope glycoproteins.

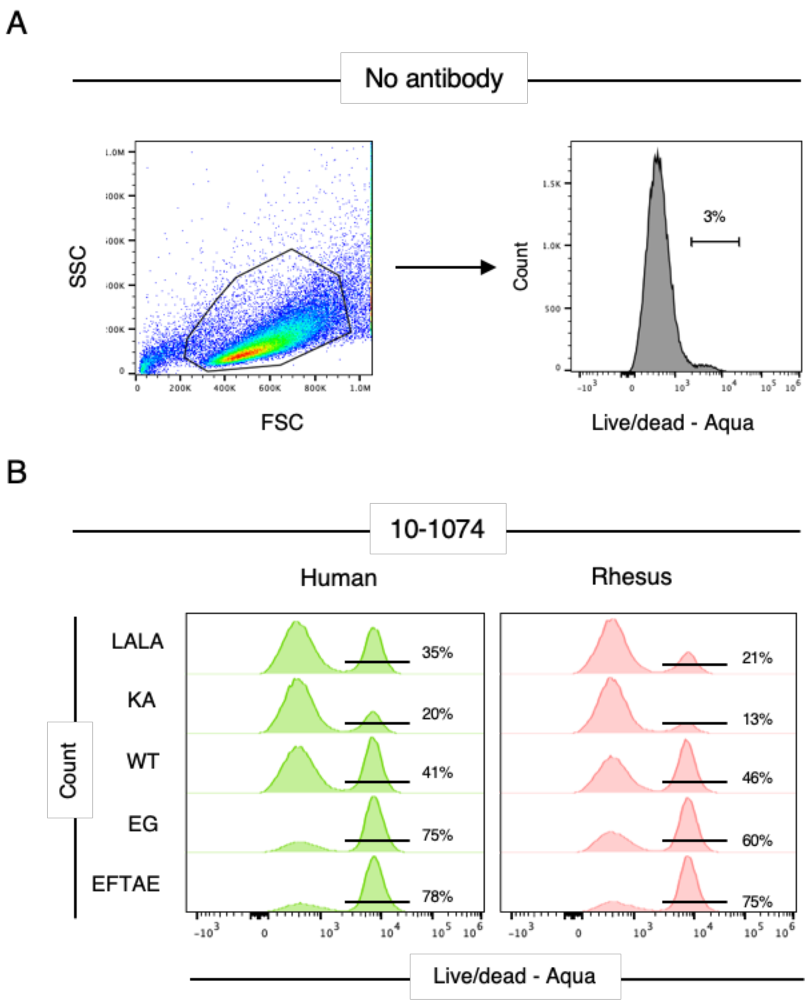

Supplemental Figure 4: Gating strategy for lytic activity of Fc-engineered antibodies. A. The FSC/SSC gate was drawn to include the Raji cells. The gate for live dead aqua stain was set using the information for no antibody control. **B**. Histograms for live dead staining for one of the human serum sample, and one of the rhesus serum sample for 10-1074 wild type and mutants.

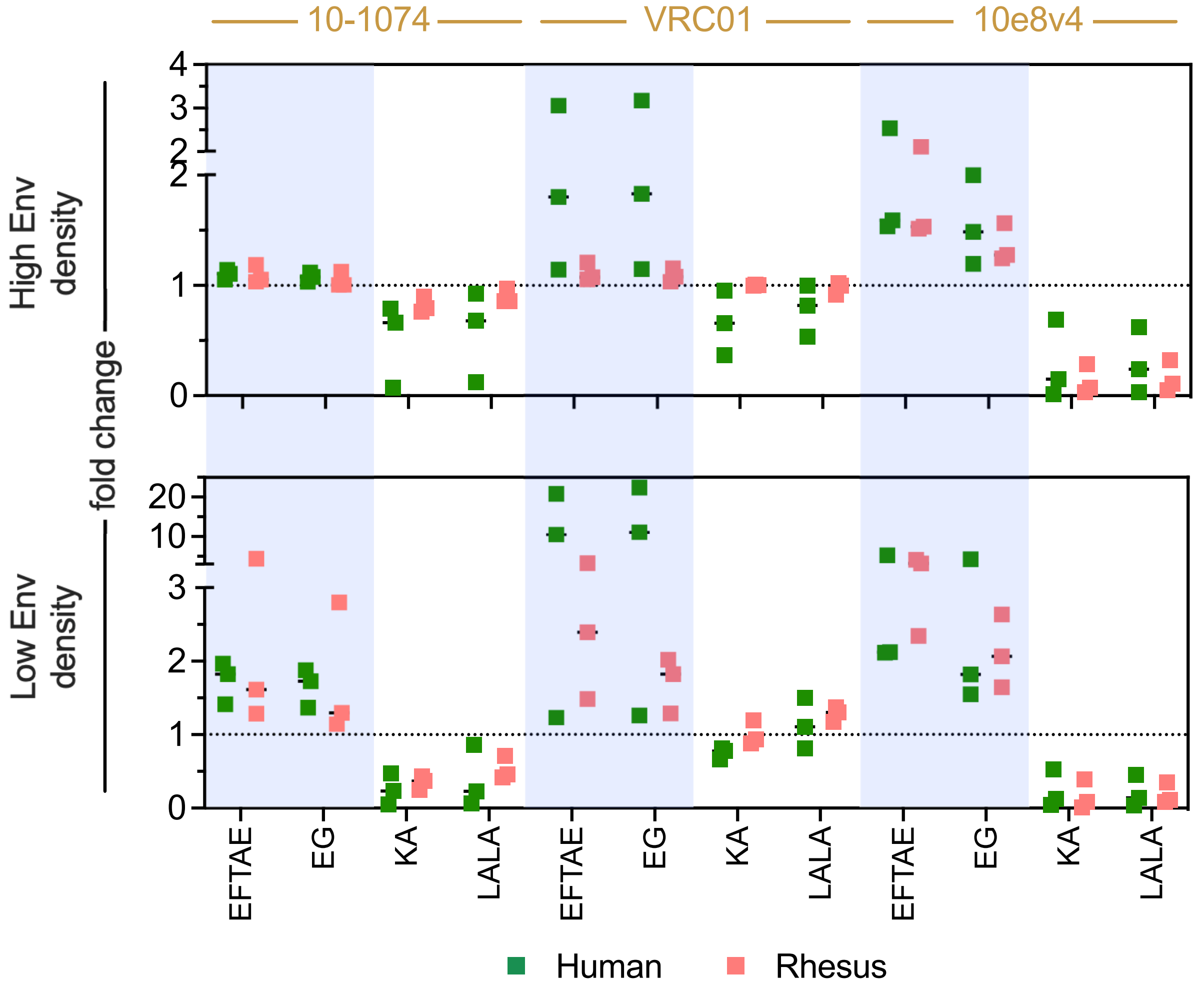

Supplemental Figure 5: Fold change of lytic activity of Fc engineered antibodies. Fold change of lytic activity of Raji cells expressing high (top) and low (bottom) levels of HIV-1 envelope by Fc engineered antibody designed to increase (EFTAE, EG; blue shading) or decrease (KA, LALA) complement activity wrt wild type antibody using serum from human (green, n=3) and rhesus (pink, n=3).

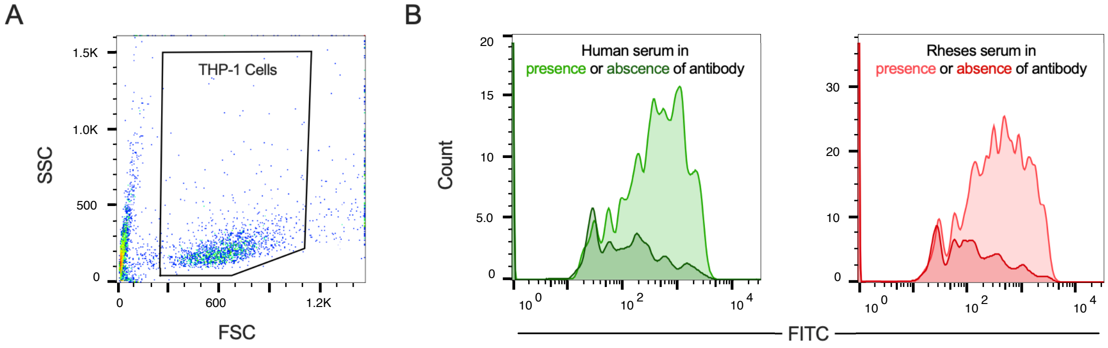

Supplemental Figure 6: Gating strategy for Complement-aided Antibody-dependent Phagocytosis (C'ADCP). A. The FSC/SSC gate was drawn to include the THP-1 cells. **B**. Histograms for phagocytosis staining for one of the human serum sample, and one of the rhesus serum sample in presence and absence of bnAb 10-1074.

Supplemental Table 1. Study Cohort. Sample size and donor age in years for human and rhesus samples used in the study.

	Human (n=90)		Rhesus (n=72)	
Median age	42		5.3	
Average age (s.d.)	43.83 (14.89)		7.15 (4.38)	
	Females (n=45)	Males (n=45)	Females (n=40)	Males (n=32)
Median age	42	40	6.25	4.95
Average age (s.d.)	44.11 (14.67)	43.56 (15.27)	6.9 (3.46)	7.46 (5.36)

Supplemental Table 2. Antigens used in multiplex assay.

Antigen	Catalogue No.	Source
A.UG37 gp140	ARP-12063	NIH HIV Reagent Program, Division of AIDS, NIAID, NIH
B.SF162 gp140	ARP-12026	
B.6240 gp140C	ARP-12572	
B.YU2 gp140 trimer*	ARP-12133	
B.9021 gp140C	ARP-12575	
B.JFRL gp140CF	ARP-12573	
B/C.CN54 gp140	ARP-12064	
C.1086 gp140C	ARP-12581	
D.UG21 gp140	ARP-12065	
F.BR29 gp140	ARP-12066	
M.CON-S gp140 CFI	ARP-12577	

*Plasmid was sourced and protein was produced by transient transfection of HEK293 Expi cells (Gibco™, A14635).
